# An RFID Based Smart Feeder for Hummingbirds

**DOI:** 10.3390/s151229886

**Published:** 2015-12-16

**Authors:** Vicente Ibarra, Marcelo Araya-Salas, Yu-ping Tang, Charlie Park, Anthony Hyde, Timothy F. Wright, Wei Tang

**Affiliations:** 1 Klipsch School of Electrical and Computer Engineering, New Mexico State University, Las Cruces, NM 88003, USA; vibarra@nmsu.edu; 2 Department of Biology, New Mexico State University, Las Cruces, NM 88003, USA; maraya@nmsu.edu; 3 Manufacturing Technology and Engineering Center, New Mexico State University, Las Cruces, NM 88003, USA; ytang@nmsu.edu (Y.T.); cpark@nmsu.edu (C.P.); ahyde@nmsu.edu (A.H.)

**Keywords:** behavioral monitoring, electrical mechanical co-design, hummingbird feeder, interdisciplinary application, passive ID tag, RFID

## Abstract

We present an interdisciplinary effort to record feeding behaviors and control the diet of a hummingbird species (*Phaethornis longirostris*, the long-billed hermit or LBH) by developing a Radio Frequency Identification (RFID) based smart feeder. The system contains an RFID reader, a microcontroller, and a servo-controlled hummingbird feeder opener; the system is presented as a tool for studying the cognitive ability of the LBH species. When equipped with glass capsule RFID tags (which are mounted on the hummingbird), the smart feeder can provide specific diets for predetermined sets of hummingbirds at the discretion of biologists. This is done by reading the unique RFID tag on the hummingbirds and comparing the ID number with the pre-programmed ID numbers stored in the smart feeder. The smart feeder records the time and ID of each hummingbird visit. The system data is stored in a readily available SD card and is powered by two 9 V batteries. The detection range of the system is approximately 9–11 cm. Using this system, biologists can assign the wild hummingbirds to different experimental groups and monitor their diets to determine if they develop a preference to any of the available nectars. During field testing, the smart feeder system has demonstrated consistent detection (when compared to detections observed by video-recordings) of RFID tags on hummingbirds and provides pre-designed nectars varying water and sugar concentrations to target individuals. The smart feeder can be applied to other biological and environmental studies in the future.

## 1. Introduction

Recent technological advances are promoting increasing numbers of interdisciplinary studies involving intelligent sensor applications. In ornithology and the poultry industry, there is strong interest in data collecting systems to study the behavior of birds. General expectations of such systems include low cost, long detection range for some predefined event, easy implementation, and low maintenance. Moreover, an intelligent system should also be able to process data from available sensors, and perform certain actions based on the received data. Such systems can greatly reduce labor, cost, and provide novel insights into animal behavior and responses to environmental change. With these design expectations, in this paper, we demonstrate a Radio Frequency Identification (RFID) based smart feeder system for hummingbirds.

RFID systems have been widely used in agriculture [1,2,3], ecology [4,5,6], and especially ornithology [7,8,9] studies. It has been proposed or demonstrated that RFID systems can be used in monitoring foraging of free-living birds [10], detecting disease [11], recording mass of birds [12], and studying their social behavior [13]. In current solutions, RFID systems are usually used to automatically record events when the target bird is present at a certain location (*i.e*., a feeder or nest) with the RFID reader in the immediate neighborhood of the target location. In the typical application, a passive integrated transponder (PIT) tag is mounted to the target bird. When the bird approaches the RFID reader, the reader records the time/duration of the visit. A more extensive review of recent RFID projects can be found in [14]. These systems have potential to perform a predefined algorithm and operate certain equipment when the visit is detected.

Currently, some systems have implemented networking and logic circuits [6], but, to our knowledge, systems equipped with mechanical devices, *i.e*., a servo, are not widely available or documented (but see [4,5]). Some systems still require handheld recording via a camera to assist the observation [2] due to their lack of automation and control. As a result, certain observations are difficult to perform in natural/semi-natural environments because of limited resources for data logging, and the lack of ability to experimentally control diets for the birds in question. Thus, there is potential to greatly enhance the performance and functionality of RFID based systems by embedding processing algorithms and mechanical devices.

In this paper, we focus on designing smart feeders to automatically record (and eventually regulate) the diet for individual hummingbirds presented with a selection of different nectar diets. Ultimately, the long-term behavior of the hummingbirds and their choices will offer insight to their cognitive ability. We targeted the feeder because feeding ecology has been a key factor in the evolution of morphology, behavior, and life history traits in birds generally, and the hummingbird in particular [15,16]. The cues and signals used to locate food have shaped animal sensory systems and cognitive abilities [17,18], and the spatial and temporal distribution of food resources has promoted the diversification of foraging strategies [19] and mating systems [20]. Hence, studying the relation between animals and their feeding resources is critical to understanding their ecology and evolution. Hummingbirds are ideal candidates to study different aspects of foraging [21] and have evolved unusual morphological [22] and physiological traits [23], to exploit nectar-producing flowers [24]. They are also easily attracted to artificial feeders, facilitating observational and manipulative studies. Thus, an RFID based smart feeder for hummingbirds can be a very useful tool for studying foraging-related traits by improving the ability to automatically manipulate feeding conditions and individualize treatments in the field.

This paper is organized as follows. In Section 2, system architecture and subsystem design are presented. Section 3 describes the experiment of a preliminary field study with the recorded data. Section 4 summarizes the system performance.

## 2. System Design and Implementation

**Figure 1:**
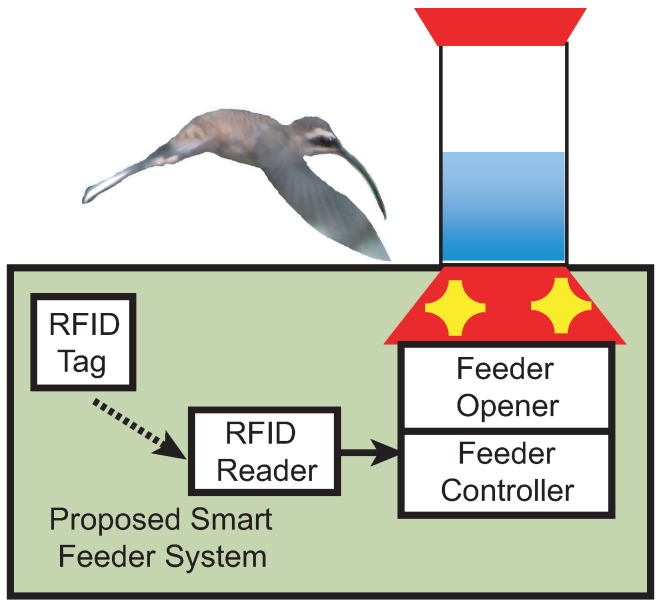
Proposed smart feeder system.

The smart feeder is designed to provide food for selected hummingbirds or to monitor the diet of a hummingbird. The operation of the smart feeder is determined by the biologist and their experimental need. The elements of the proposed system are shown in Figure 1. The target hummingbirds each have a PIT tag affixed to their body. In one study, when a hummingbird approaches the feeder, the RFID reader detects the tag ID and the feeder controller decides whether the feeder should be opened, based on diet regulation and the algorithms which dictate the predefined experiment. A second study provides data logging for each visit to a feeder. In both circumstances, the RFID reader records the PIT tag number as well as the time of the event for further biological studies. In this section, we introduce the design considerations and implementation details of the proposed system.

Because the deployed system is expected to interact with hummingbirds, there are several design concerns that must be addressed. First, we want to minimize the influence of the extra devices on the target hummingbirds during the feeding process. Specifically, we require tags of minimal mass and size that will not affect the behavior of the hummingbirds. General guidelines call for tags that weigh no more than 3%–5% of a hummingbird’s body weight [12]. Second, we want to prevent the feeding mechanism from conditioning the feeding habits of hummingbirds. For instance, the size of the RFID reader and antenna must have a minimal visual footprint so the hummingbirds are not afraid of the feeder and are not provided visual cues that would interfere with the study. Third, it should ensure that the hummingbirds are not harmed during the experiment. The feeder should not close while the hummingbirds are feeding, and this requirement is addressed by several fail-safes. Fourth, we want to reduce the false detection by optimizing the detection range. Fifth, there are size, weight, and power (SWaP) requirements that are imposed by conditions of deployment in the field setting. In particular, the feeder should tolerate high humidity working conditions since they are deployed in a rainforest environment. Finally, we want to reduce the cost of the system to enable future mass deployment in the field.

**Figure 2:**
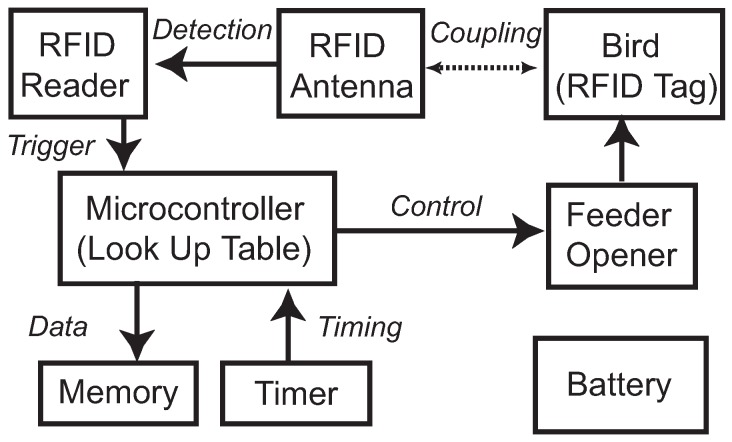
System schematic of the smart feeder system.

**Figure 3:**
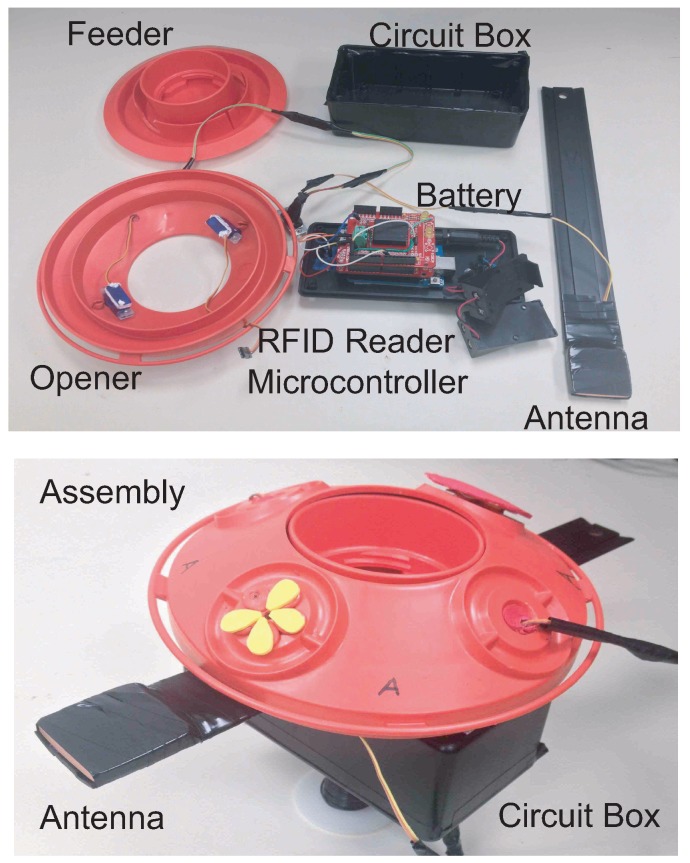
System assembly of the smart feeder. (**Top**) all components with the circuit box; (**Bottom**) final assembly.

The proposed system consists of three subsystems: a microcontroller subsystem, an RFID subsystem, and a mechanical feeder opener. The RFID reader monitors the environment through the antenna and searches for PIT tags nearby. When a tag is detected, the ID number is recorded by the microcontroller. The microcontroller along with the supporting electronics, including a clock module and a memory module, records ID number and timing of each detection. The microcontroller also makes a decision based on predefined algorithms, and regulates the response of the feeder opener. Figure 2 presents the detailed system schematic. Figure 3 shows the completed assembly of the smart feeder system.

### 2.1. Microcontroller Subsystem

In this section, we describe operation of the microcontroller and its peripheral devices. The microcontroller serves as the main processing unit for the smart feeder. The primary objectives are control of the RFID subsystem, the mechanical opener, the timing unit, and the memory card. All circuits are powered by a 5 V power supply regulated from the 9 V batteries by the Arduino platform. The communication between the microcontroller and its peripheral devices uses universal-asynchronous-receiver/transmitter (UART) serial protocol to save power.

**Figure 4:**
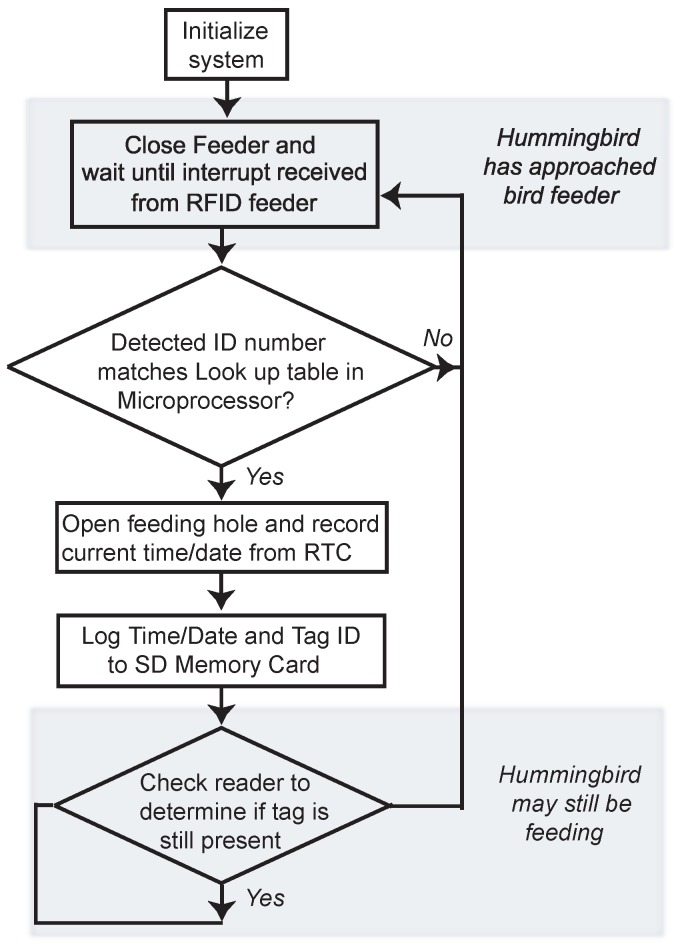
Microcontroller software flow chart.

The microcontroller operates according to the algorithmic flow chart in Figure 4 and is programmed with the C language. Given the experimental study of diet regulation (*versus* diet monitoring), the microcontroller functions as follows. Initially, the smart feeder is powered on with the feeding orifice in the closed position (initialization stage). Next, the microcontroller monitors the RFID sensing module, which is continually searching the immediate environment for the presence of an RFID tag. If a tag disrupts the magnetic field, the microcontroller registers the event and proceeds to compare the detected RFID tag number with a look-up table (LUT) that is written in the microcontroller’s memory to determine whether the feeder orifice should be open. In a simplified operation, hummingbirds are granted or denied access to feeders according to the LUT. The detected event is then logged into the Secure Digital (SD) memory card; specifically, the time and tag ID number are stored. Depending on the study design, the hummingbirds’ diet can also be individually regulated by controlling access to particular feeders in an array.

**Figure 5:**
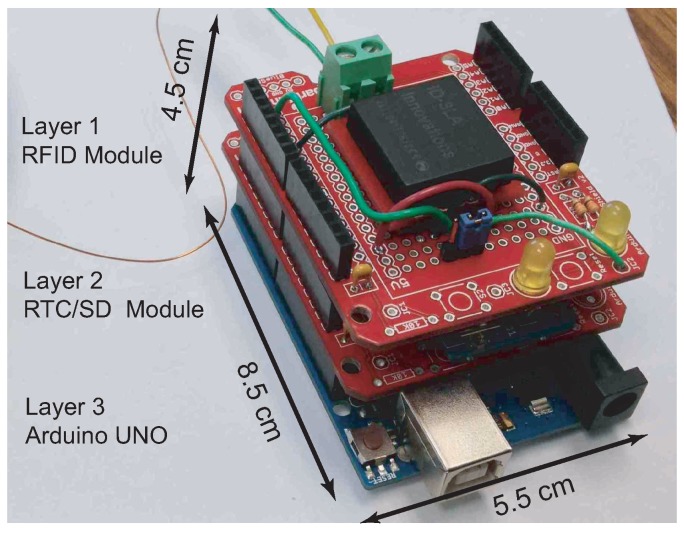
Electrical subsystem in the smart feeder system. The three layer board hosts RFID reader, real-time clock (RTC)/SD module, and the Arduino microcontroller module.

In the event that the bird remains feeding, it is protected from being hurt by the feeder closing on its bill via several mechanisms. Every experiment is conducted with the batteries charged to their maximum capacity which ensures that the feeders will operate as expected during the experiment. Each experiment has a duration that is significantly less than the system battery life. These two conditions ensure that the system can always command the servo to a defined state and monitor the presence of a bird. The microcontroller also continually monitors the past/present magnetic field state in order to ensure the bird has left the area. The servo can only open when a tag is confirmed and can only close when the bird has been verified to leave the feeder. These measures protect the birds’ beak from being damaged by the mechanical arm that regulates access to the feeder. The microcontroller is supported by a real-time clock (RTC) and SD card to record the approximate local time of each feeding event. The microcontroller, RTC, and the memory card were selected from off-the-shelf components by considering their physical size, weight, power consumption and performance. The AtMega328P on an Arduino board was selected as the microcontroller for ease of portability and due to the abudant references available (which allows biologists to modify the system in a worst-case scenario). The Maxim DS1307 was selected as the RTC since it has a life expectancy of five years on a single 3 V 25 mAh coin cell battery. Lastly the storage solution is a single 8 GB microSDHC memory card, which granted the ability to log a significant amount of data (which is all text based) and is easy to interface with. The assembly of electrical subsystem is shown in Figure 5.

### 2.2. RFID Subsystem

In this section, we present design of the RFID subsystem. The RFID subsystem consists of the RFID reader, RFID antenna and PIT tag. An RFID reader detects the nearby PIT tag via the RFID antenna. Detection begins with the tag being excited by a signal at the frequency of interest. The tag (which is entirely passive) then uses the incident energy to create a modulation effect by switching a load on/off. The switching follows a pattern based on each tag ID and is used as a means of differentiating each bird.

Given the anticipated application, the RFID tag size, weight, and detection range are the most important considerations due to the restrictions imposed by the long-billed hermit (LBH). A 125 kHz RFID system was selected due to the miniature size of its PIT tag. In general, the detection range is determined by the size of the antenna and the supply current to the antenna (in this case, it is preset by the RFID reader). In this project, we required a 5–15 cm detection range and the size of the antenna had to be small enough to be implemented on the feeder, which has a diameter of 40 cm. Based on the above considerations, the ID Innovations ID-3LA reader module was selected as the RFID reader, which requires an external antenna. The reader module provides 125 mA of fixed output current to the external antenna. Optimization of the detection range is thus a function of the antenna and is achieved by engineering the antenna geometry and impedance.

**Figure 6:**
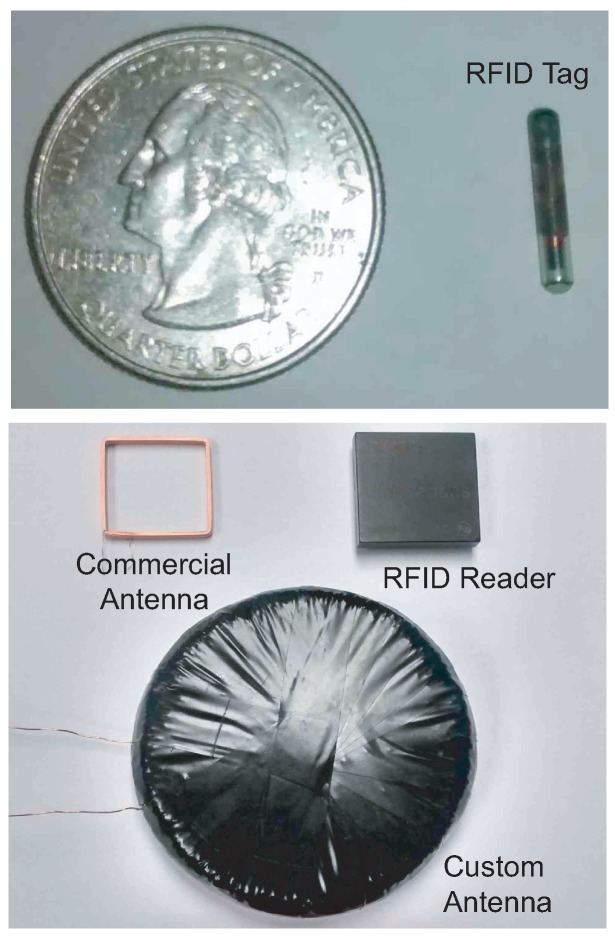
PIT tag (**top**) RFID reader, and reader antennas; (**bottom**) in the proposed smart feeder system.

We compared different antenna solutions for the RFID system. The antenna selected for the system was chosen after a trade study that addressed risks pertaining to performance and manufacturability with respect to the RFID transceiver. Three antennas were initially considered. The first was the ID-Innovations ID-20 transceiver. This module is similar to the ID-3LA with the exception of including an internal antenna. The second antenna was a commercial machine-wound rectangular coil. Lastly, a custom hand-wound circular coil was fabricated to achieve the manufacturer recommendation for the ID-3LA. Although the custom antenna achieved longer detection distance compared to the other two antennas, a reliability concern led us to choose the commercial antenna for the smart feeder, which had a longer detection range than the internal antenna of ID-20. The reliability concern stemmed from the fact that each hand-wound antenna would have varying characteristics when compared to machine-wound (which produces a consistent coil with minimally varying characteristics). The RFID tag, reader, and antennas are shown in Figure 6.

### 2.3. Power Budget

The smart feeder system was built with the intention of assisting New Mexico State University (NMSU) biologists with a separate study of LBH cognitive ability. The study/experiment put forth by the NMSU researchers required an observation period of two hours at different times of the day. This figure was used as a requirement for the power budget for the system given that the system would need to operate for two hours at a time. The system was found to consume 53 mA of current when in an idle state (*i.e.*, when no birds are present). When a bird visits the feeder, the system demands additional current in order to move the lever-arm of the servo. This current draw was determined to be 155 mA consumed over a duration of 450 mS (open and close). A generous estimate is to expect a bird to visit the feeder once per minute. Thus, given that the experiment is executed in two-hour segments, the equivalent duration of the current draw is 54 min (or 0.9 h). The total energy requirement is calculated to be 245.5 mAh. Given that the system operates off of two 9 V batteries in parallel with a total capacity of 600 mAh, the system is able to meet the minimum operating time of two hours per experiment. Additional operation time can be achieved by adding more batteries.

### 2.4. Mechanical Feeder Opener

The servo-controlled feeder opener is another key component of the smart feeder. The opener contains a servo controlled by the microcontroller, and the opening lever arm controlled by the servo. Both the servo and opening arm are installed on the feeding tray of an off-the-shelf hummingbird nectar feeder (16 oz Clean Feeder, Dr. JB’s Hummingbird Products (Songbird Essentials, Mexico, MO, USA)). The major requirement for the mechanical design was ensuring the lever arm could move freely in grooves machined underneath the standard flower-shaped openings. Minimization of the noise created by the movement of the lever-arm during the servo operation was also critical because both of these factors could provide unwanted cues for the birds which would ultimately compromise the study of the biologists. These requirements (seamless movement and minimal noise) required custom-machining of the feeder.

**Figure 7:**
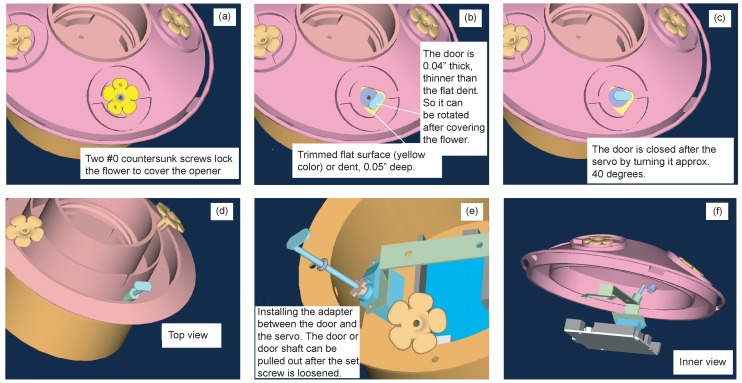
Computer aided design models of installing custom feeder opener on an off-the-shelf hummingbird feeder. The main steps include (**a**) modifying the flower opening; (**b**) resurfacing the area beneath the flower opening; (**c**) and (**d**) installing the servo operated door; (**e**) connecting the door to the servo motor, and (**f**) installing the servo motor underneath the feeder.

CAD tool Pro/ENGINEER was used to build a 3D model of the feeder. The trapdoor design reduces the risk of clogging the opener due to the nectar. CAD modeling defined the size restrictions of the servo. In this design, the POLOLU Corp 1053 sub-micro servomotor was selected, which had a torque of 0.056 N·m. This was sufficient for operating the smart feeder. With the servo dimensions known, the model was transferred to a four-axis computer numerical control (CNC) mill using a CAM software CAMAX.

The design sketch and details of the mechanical drawing are shown in Figure 7. The custom machining of the feeder was performed at Manufacturing Technology and Engineering Center (MTEC) in New Mexico State University.

## 3. Experiment Result

### 3.1. RFID Tag and Mounting

**Figure 8:**
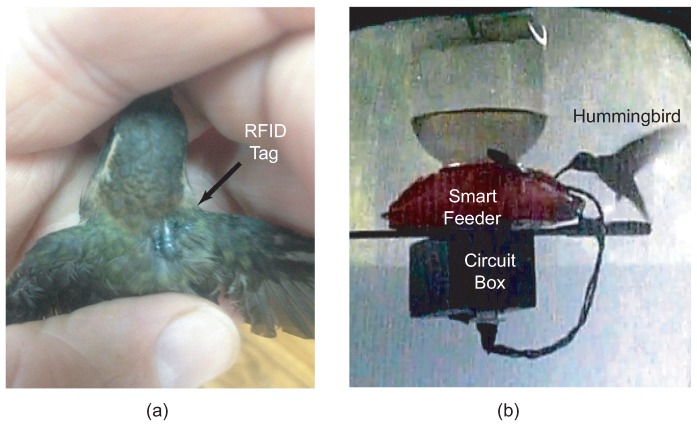
(**a**) PIT tag adhered to hummingbird; (**b**) Hummingbird consuming nectar food from the proposed RFID smart feeder.

The PIT tag used in the experiment is an industry standard glass capsule. The cylindrical capsule has an approximate volume of 12 × 2 × 2 mm${}^{3}$, a mass of 0.095 g and is resonant at 125 kHz. While several factors contributed to the selection of the tag, the deciding factors are the tag’s mass, availability, and price. Tag mounting procedure is another key factor in the experiment. Instead of implantation, leg-mounting, or leg-bands, (all of which are typical in the literature), we find that directly attaching the PIT tag under the feathers worked very well. The tag was adhered using a non-toxic adhesive by embedding the tag within the feathers of the LBH, as shown in Figure 8a. This mounting process is easier than fabricating leg-bands for each bird. It also provides a consistent method of detection when tested with RFID reader.

### 3.2. Field Experiment

**Figure 9:**
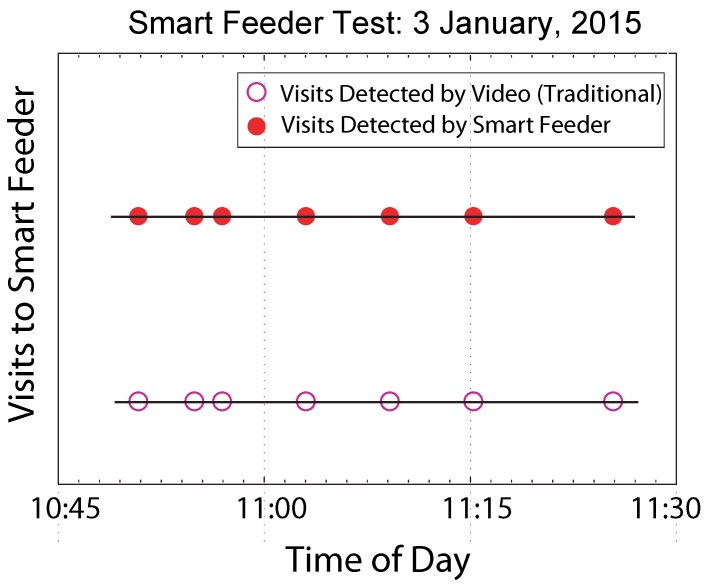
Comparison of detected LBH visits to a smart feeder based on video recording and RFID detection for a single trial.

The completed system was tested in the field at La Selva Biological Station in Costa Rica with wild LBH. The hummingbirds were fed manually for several hours to allow them to adapt to the feeders. Feeding manually required placing the beak of the LBH into the feeder until it was observed that the LBH became aware of the presence of the nectar. Once the hummingbirds were observed feeding unassisted, then the hummingbirds were fitted with the PIT tags. Testing consisted of monitoring the feeder with a digital camera as a means of confirming visits to the smart feeders. For the study presented in this paper, a single bird with a single or multiple feeders was studied in a private enclosure, thus removing the risk of an untagged bird complicating the experiment. Figure 8b shows a hummingbird drinking nectar from the smart feeder inside the enclosure. During the testing, the smart feeders were proven to provide a reliable means of both detecting the presence of the LBH and offering nectar. Figure 9 plots visits to the smart feeder *versus* time of day as observed by a video camera and the smart feeder. These data demonstrate that the smart feeder can provide an effective, efficient, and potentially more accurate alternative to monitoring behavior with human observers or a video camera. Both video and direct observations showed no evidence that the sound or movement of the servo alarmed the hummingbirds.

## 4. Conclusions

In this paper, we demonstrated an RFID based smart feeder system (summarized in Table 1) which can be used as an effective means of monitoring hummingbirds and performing experiments dealing with cognitive ability. The system has demonstrated the ability to monitor hummingbirds in a semi-natural environment in a rainforest and to facilitate novel experiments monitoring and manipulating diet. The system presented can easily be used by biologists to conduct research that has proven to be difficult to automate. The system is also easily adapted to similar scenarios; however, the primary use should revolve around regulating the feeding of birds. To our knowledge, this is the only demonstration of the ability to perform noninvasive experimentation and individualized manipulation of a bird’s diet which grants biologists the ability to perform long-term studies based on feeding habits (comparison of similar systems are summarized in Table 2). More behavioral studies with the smart feeder are planned for the future.

**Table 1:** Measured parameters of the proposed radio frequency identification (RFID) smart feeder.

Parameters	Value
RFID Tag size	1.3 × 0.8 × 0.6 mm
RFID Tag weight	0.095 g
RFID Antenna range	9–11 cm
RFID Antenna size	3.6 cm × 3.6 cm
System power supply voltage	9 V
logging timing resolution	1 s
Feeder opener response time	0.5 s
RFID frequency	125 kHz

**Table 2:** Comparison of recently reported RFID based ornithology applications.

	[6] 2013	[2] 2009	[12] 2014	This Work 2015
Target Bird	Seabird Shearwater	Emu	Humming-bird	Humming-bird
RFID Frequency	13.56 MHz	915 MHz	125 kHz	125 kHz
Detection Range	28 cm	100 cm	1–9 cm	10 cm
Application	Data Logging	Data Logging	Data Logging	Data Logging & Diet control
Mechanical Devices	No	No	No	Yes
On device signal processing	No	No	No	Yes
